# SHEA statement on antibiotic stewardship in hospitals during public health emergencies

**DOI:** 10.1017/ice.2022.194

**Published:** 2022-11

**Authors:** Tamar F. Barlam, Mayar Al Mohajer, Jaffar A. Al-Tawfiq, Antonie J. Auguste, Cheston B. Cunha, Graeme N. Forrest, Alan E. Gross, Rachael A. Lee, Susan K. Seo, Kathryn N. Suh, Stacy Volk, Joshua K. Schaffzin

**Affiliations:** 1 Department of Medicine, Boston University School of Medicine, Boston, Massachusetts, United States; 2 Boston Medical Center, Boston, Massachusetts, United States; 3 Department of Medicine, Baylor College of Medicine, Houston, Texas, United States; 4 Baylor St. Luke's Medical Center, Houston, Texas, United States; 5 Department of Medicine, Indiana University School of Medicine, Indianapolis, Indiana, United States; 6 Johns Hopkins Aramco Healthcare, Dhahran, Saudi Arabia; 7 Department of Medicine, University of Pittsburgh, Pittsburgh, Pennsylvania, United States; 8 Infectious Disease Connect, Pittsburgh, Pennsylvania, United States; 9 Department of Medicine, Alpert Medical School of Brown University, Providence, Rhode Island, United States; 10 Rhode Island Hospital, Providence, Rhode Island, United States; 11 Department of Infectious Diseases, Rush University Medical Center, Chicago, Illinois, United States; 12 Department of Practice, University of Illinois at Chicago College of Pharmacy, Chicago, Illinois, United States; 13 Department of Medicine, University of Alabama at Birmingham, Birmingham, Alabama, United States; 14 Birmingham VA Medical Center, Birmingham, Alabama, United States; 15 Department of Medicine, Weill Cornell Medical College, New York, New York, United States; 16 Memorial Sloan Kettering Cancer Center, New York, New York, United States; 17 Department of Medicine, University of Ottawa, Ottawa, Ontario, Canada; 18 Intermountain Healthcare Lutheran Medical Center, Wheat Ridge, Colorado, United States; 19 Department of Pediatrics, University of Cincinnati, Cincinnati, Ohio, United States; 20 Cincinnati Children’s Hospital Medical Center, Cincinnati, Ohio, United States

## Introduction

This statement addresses the inappropriate antibiotic prescribing occurring during the coronavirus 2019 pandemic (COVID-19) that has exacerbated another urgent public health crisis: antibiotic resistance in bacterial and fungal pathogens.^1–3^ Ramifications of overprescribing have led to infections with multidrug-resistant organisms (MDROs) such as extended-spectrum β-lactamase–producing gram-negative bacteria and methicillin-resistant *Staphylococcus aureus* (MRSA).^4^ These infections complicate patient treatment, prolong hospital stays, and lead to worse outcomes.^5^

The emergence of severe acute respiratory virus coronavirus 2 (SARS-CoV-2), has resulted in an unprecedented pandemic with >535 million cases worldwide and >6 million deaths as of June 2022.^6^ The United States has reported >85 million cases and >1 million deaths,^7^ surpassing the number caused by the 1918 influenza pandemic.^8^ The pandemic has reinforced prior analyses, which identified numerous vulnerabilities, including inconsistent funding for US public health preparedness and response that contributed to inadequate resources for treatment, testing, and contact tracing, and breakdowns in the supply chain for essential healthcare equipment.^9–11^

As a new disease, COVID-19 led to rapidly evolving information, particularly early in the pandemic. Patients with COVID-19 can present with severe illness and clinical and laboratory findings suggestive of bacterial coinfection. Because bacterial coinfection seen with other viral respiratory infections—particularly influenza—may increase a patient’s morbidity and mortality,^12^ the same concern was present for COVID-19. Consequently, inappropriate antibiotic use has increased.^4,13,14^

**Fig. 1.**
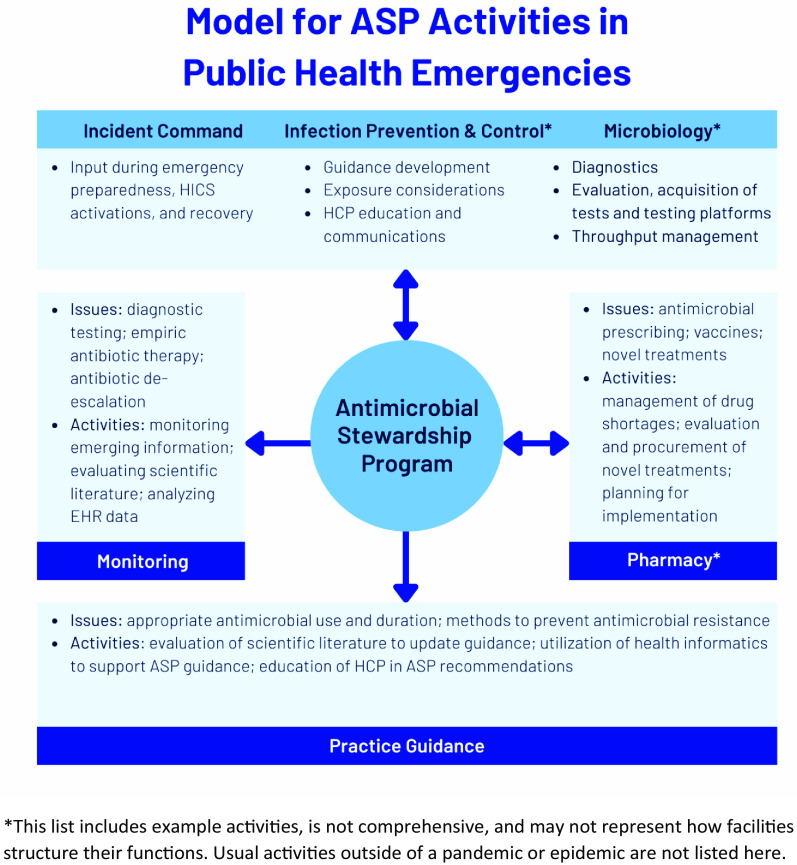
Model for ASP Activities in Public Health Emergencies.

In this paper, we discuss the conditions of the COVID-19 pandemic that resulted in inappropriate antibiotic use in adult hospitalized patients and approaches to improve practice for the next public health emergency, as well as the central role of antibiotic stewardship programs (ASPs) in pandemic response (Fig. 1). Table 1 provides an overview of the acronyms used in the manuscript. Table 2 summarizes the recommendations.

**Table 1.** Acronyms

• APIC: Association for Professionals in Infection Control and Epidemiology
• ASP: antibiotic stewardship program
• ASPR: Office of the Assistant Secretary for Preparedness and Response
• CAP: community-acquired pneumonia
• CDC: Centers for Disease Control and Prevention
• CRP: C-reactive protein
• CT: computed tomography
• EBM: evidence-based medicine
• EHR: electronic health record
• EMP: emergency management program
• EOP: emergency operations plan
• HCP: healthcare personnel
• HICS: hospital incident command structure
• ICU: intensive care unit
• IDSA: Infectious Diseases Society of America
• MDRO: multidrug-resistant organism
• MRSA: methicillin-resistant staphylococcus aureus
• PCR: polymerase chain reaction
• SHEA GLC: SHEA Guidelines Committee
• SHEA: Society for Healthcare Epidemiology of America
• SIDP: Society of Infectious Diseases Pharmacists

**Table 2.** Recommendations

Question	Recommendation(s)
How can facilities and HCP improve antibiotic prescribing during a pandemic or epidemic?	HCP should limit initiation of antibiotics—particularly broad-spectrum agents—for patients in acute care settings with high pre-test probability for a viral infection, such as SARS-CoV-2 or influenza, even if there is lack of readily available and accurate diagnostics.
What diagnostic testing is appropriate for patients admitted to the hospital?	HCP may perform inflammatory marker tests at baseline, particularly in critically ill patients, including C-reactive protein, lactate dehydrogenase, D-dimer, serum ferritin, and high-sensitivity troponin. HCP should repeat laboratory testing only to the extent that it provides actionable clinical data. HCP can monitor CRP if clinically indicated to inform respiratory viral treatments. For COVID-19, CRP monitoring informed use of biologic agents such as tocilizumab or baricitinib. HCP should not use inflammatory markers as the basis for initiation of antibiotics or antifungal agents, as they may not be indicative of bacterial coinfection. HCP should not use procalcitonin routinely to aid in the decision to initiate antibiotics. HCP will need to monitor the relationship of inflammatory markers to infection in future infectious epidemics and pandemics. HCP should not obtain bacterial cultures or respiratory multiplex PCR tests for patients who do not have indicators consistent with bacterial infection, particularly those with stable clinical status in a non-ICU setting.
When should antibiotics be considered for patients during respiratory viral pandemics and epidemics?	It is important for HCP to identify patients who require empiric antibiotic treatment, despite low rates of coinfection on admission for the COVID-19 and prior respiratory viral pandemics. If treated at the time of hospital admission, HCP should prescribe antibiotics recommended in guidelines for CAP for patients presenting from the community with co-infection. Antibiotics for pathogens such as *Pseudomonas aeruginosa* seen more typically in healthcare-associated infection are not recommended. HCP may consider coinfection with other viruses, such as influenza. If influenza is diagnosed, addition of therapy, such as oseltamivir, may be indicated. HCP may consider antibiotics, including broad-spectrum coverage, for patients hospitalized for >48–72 hours who are at greater risk for HAIs and MDROs, when there are new clinical signs that are consistent with healthcare-associated bacterial or fungal infection.
What microbiologic and radiographic diagnostic tests are appropriate if antibiotics are started for patients with possible bacterial co-infection?	Before initiating antibiotics, HCP should attempt to obtain a microbiologic diagnosis. HCP should limit respiratory multiplex PCR tests to ICU patients and patients who require broad-spectrum antimicrobial therapy. HCP should restrict repeat microbiologic testing to changes in a patient’s clinical status. HCP should perform a nasal MRSA swab for patients started on anti-MRSA treatment. HCP should not perform routine testing for fungal infection in the absence of a clinical presentation that raises that concern. HCP should review the necessity of antibiotics within 48 to 72 hours as test results become available and should de-escalate or discontinue antibiotic therapy based on those results and clinical response. Procalcitonin results may aid the de-escalation or discontinuation of antibiotic treatments. HCP should obtain chest radiographs to assess for the extent of lung involvement, but daily repeat studies are not indicated. Use of computed tomography (CT) of the chest should be reserved for circumstances in which results of the CT may result in a change in clinical management (eg, pulmonary embolus).
What is the role of the ASP in a pandemic or epidemic?	ASPs should monitor emerging information and updates to national and international guidelines that are relevant to antibiotic prescribing, revise clinical recommendations relevant to antimicrobial use, and educate frontline HCP to support appropriate antibiotic stewardship.

## Intended use

This consensus statement advises hospitals and healthcare personnel (HCP) on ways to improve antimicrobial stewardship and antimicrobial use during infectious disease crises, including outbreaks, epidemics, and pandemics. No guideline, expert guidance, or white paper can anticipate all situations, and this document is not meant to be a substitute for individual judgment of qualified professionals or oversight panels.

## Scope and terminology

The statement addresses infectious disease disasters as they affect hospitals. It is framed within the context of lessons learned during the COVID-19 pandemic. Natural disasters, biochemical disasters, terrorism, or other noninfectious events are outside the scope of this document.

The authors use the term “coinfection” to refer to individuals who present to a healthcare facility and (1) are confirmed to be infected with the outbreak, epidemic, or pandemic pathogen and (2) are infected with a community-acquired bacterial, fungal, or viral infection. “Healthcare-associated infection” refers to bacterial, fungal, or viral infection that occurs in a patient at least 48 hours after hospital admission for infection tied to an outbreak, epidemic, or pandemic.

## Methods

Unlike the expert guidance format used for other recommendations documents that are sponsored by the Society for Healthcare Epidemiology of America (SHEA), this document is not based on a systematic literature review. SHEA employed a consultant medical librarian to develop a search strategy encompassing antimicrobial stewardship, antimicrobials, COVID-19, and related terms. The search was run on PubMed for the period from December 1, 2019, to August 17, 2021. The authors screened the abstracts in the Covidence abstract management service (Melbourne, Australia) and reviewed full-text articles as PDFs.

This document was reviewed by the SHEA Guidelines Committee (GLC), the SHEA Publications Committee, the SHEA Board of Trustees, the Infectious Diseases Society of America (IDSA) Standards and Practice Guidelines Committee, the IDSA Board of Directors, the Pediatric Infectious Diseases Society (PIDS), the Society for Critical Care Medicine (SCCM), and the Society of Infectious Diseases Pharmacists (SIDP). This white paper was endorsed by SHEA, IDSA, PIDS, SCCM, and SIDP.

## Authors

This document was developed by a multidisciplinary panel of experts who are members of the SHEA Antibiotic Stewardship Committee and SHEA Guidelines Committee and are experts in infectious diseases and antimicrobial stewardship, as well as representatives from IDSA and SIDP. All authors are involved at their respective institutions in antimicrobial stewardship, either directly or in an advisory role.

## Lessons learned from COVID-19: Factors that influenced antibiotic prescribing

### Evolving information on clinical presentation and the risk of progression to severe illness

With any emerging infection, descriptive information is needed to understand the disease characteristics and outcomes of affected patients. As large-scale observations from early epicenters of the COVID-19 pandemic were published, it was evident that, as with other respiratory viruses, COVID-19 presents in patients on a spectrum ranging from asymptomatic carriage to fulminant disease.^15,16^ Critically ill individuals can have respiratory failure, septic shock, and/or multiorgan dysfunction. Early in the pandemic, ∼14% of patients required hospitalization and 2%–5% required a stay in the ICU.^15^ Risk factors for severe infection include immunosuppression (eg, patients with cancer, organ, or hematopoietic stem cell transplantation), obesity, or the presence of 1 or more comorbidities (eg, cardiovascular disease, chronic kidney or lung disease, diabetes mellitus).^15,17^

The categorization of the patient’s condition can be dynamic. Patients who have signs or symptoms of lower respiratory tract involvement (eg, dyspnea, infiltrates on chest imaging) often require admission and they can rapidly develop hypoxemia (oxygen saturation <94% on room air) and need supplemental oxygenation or ventilatory support—signifiers of severe disease. For hospitalized patients, a key objective has been to promptly identify and manage high-risk severe cases to mitigate poor outcomes. Clinical uncertainty^18–21^ and discomfort at not taking action for patients who continued to show signs of worsening despite supportive care, including continued fever, signs of inflammation with progressive hypoxemia, laboratory markers, and/or radiological findings of increasing disease severity,^22^ contributed to inappropriate antibiotic prescribing.^22–24^ Also, a lack of sufficient personal protective equipment (PPE) limited entry of healthcare personnel (HCP) into patient isolation rooms for frequent clinical reassessment,^11,25^ resulting in a low threshold to begin antibiotics and hesitation to withdraw treatments viewed as possibly helpful.

### Uncertainty about the COVID-19 diagnosis

Early in the pandemic, there were significant delays in reliable diagnostic tests and constrained testing capacity for SARS-CoV-2. The lack of definitive COVID-19 diagnostic tests,^26^ especially during the initial spread of cases into communities where COVID-19 incidence was still low, resulted in overtreatment for other causes of pneumonia.^27^ Physicians often felt they had to treat for bacterial pneumonia with antibiotics, despite clinical presentations consistent with COVID-19. Even when the availability and turnaround time of test results improved, viral kinetics of SARS-CoV-2 and its spread were misunderstood, leading to delays in diagnosis or misdiagnosis.^28^

Balancing the accuracy and reliability of diagnostic tests with the setting where they are used (eg, emergency departments vs outpatient urgent care) and the purpose for which they are used (eg, diagnosis vs screening personnel by testing) continues to be a challenge almost 2 years later. SARS-CoV-2 diagnostic tests vary in reliability and accuracy based on type (eg, RT-PCR assay vs antigen test), the presence or absence of symptoms, time since exposure, site of testing (nasal vs deep bronchial), and the quality of the sample.

As processing, availability of materials, and turnaround time of testing for COVID-19 improved, empiric antibacterial use also decreased.^27^ More than half of patients positive for COVID-19 had antibacterial therapy stopped within 1 day of return of a positive COVID-19 test.^27^ However, initiation of antibiotics remains high.

### Concern for bacterial coinfection at the time of admission

Historically, heightened concern for bacterial coinfection has been the norm during viral respiratory outbreaks, such as those caused by influenza or SARS-1.^29^ However, prior pandemics and epidemics due to respiratory viruses have shown varying levels of bacterial and fungal coinfection.^30^ Despite the fact that true bacterial coinfection at presentation is low for COVID-19, estimated at 3.1%–5.5%,^31,32^ the concern for bacterial coinfection has been evident and has driven antibiotic prescribing. Studies have reported broad-spectrum antibiotic use for COVID-19 patients frequently, with 60%–100% of patients receiving antibiotics on or shortly after admission.^22,24,33–35^ In addition, early in the pandemic, the media focused on reports promoting azithromycin as a potential anti-inflammatory treatment, contributing to use of that agent. Some studies conducted within the first few months of the pandemic did note reduced empiric use of antibiotics over time,^36^ but a recent multicenter cohort study still demonstrated that 56.5% of 1,705 patients with COVID-19 received empiric antibiotic therapy. Patients were more likely to receive empiric antibacterial therapy if they were older or had a lower body mass index.^27^

### Concern for healthcare-associated bacterial infection

Unlike the risk of bacterial coinfection on hospital admission, the risk of bacterial or fungal healthcare-associated infection (HAI) increases with prolonged hospitalization duration and broad-spectrum antibiotic use. This is particularly important among individuals with severe COVID-19. Multiple studies have reported HAI rates of 27% to 37% among ICU patients.^37–39^ In a study comparing COVID-19 and non–COVID-19 cases, the rate of HAIs per 1,000 ICU days was 13.76 and 7.04, respectively.^40^ Another study reported a rate of 44.7 infections per 1,000 ICU patient days.^41^ HAIs occurred in 46% of patients with COVID-19 in this study, with ventilator-associated pneumonia and bloodstream infections as the most common causes. Also, 35% of HAIs in critically ill patients with COVID-19 were caused by multidrug-resistant bacteria.^41^ HAIs are particularly concerning when caused by MDROs because they drive more frequent use of broader-spectrum antimicrobial agents.

## Diagnostic studies

### Inflammatory markers elevated in COVID-19 are not reliable indicators of bacterial or fungal infection

In COVID-19, elevated inflammatory markers may be due to immune dysregulation, persistent hyperinflammation, and cytokines, but they have been incorrectly interpreted as markers of bacterial coinfection. COVID-19, in the absence of bacterial or fungal infection, causes elevations in white blood cell count (WBC), C-reactive protein (CRP), and sedimentation rate (ESR). Although procalcitonin (PCT) was a biomarker that was shown to differentiate viral and bacterial pneumonia in prepandemic studies,^42,43^in COVID-19, it is frequently a marker of severe disease rather than bacterial coinfection.^44^ A meta-analysis of 4,911 patients from 29 studies found that WBC count >10×10^9^/L, PCT >0.5 ng/mL, and CRP >10 mg/L were risk factors for COVID-19 disease progression.^45^ Bacterial infections can also produce some of the same findings. For example, a retrospective cohort study of community-acquired pneumonia (CAP) and COVID-19 pneumonia found that CAP was characterized by high baseline WBC count (median, 12.48 vs 6.78 ×106 cells/mL), CRP (median, 133.5 vs 86.0 mg/L), and greater reduction in CRP 48–72 hours into admission (median ΔCRP, −33 vs +14 mg/L) after antibiotic use.^46^

Such complex findings contribute to antibiotic overuse. Elevation of these markers at admission to the hospital, or new elevation days into hospitalization, has been a rationale for initiating antibiotic treatments, even though most patients have these laboratory abnormalities due to the progression of the primary COVID-19.

### Microbiologic studies

Blood-culture utilization increased during the COVID-19 pandemic. In one hospital network, there was a 34.8% increase in blood-culture ordering in the second half of March 2020 compared with the first half, which was thought to be due to an increase in ordering in patients with COVID-19.^47^ The positivity yield for blood cultures were lower in patients positive for SARS-CoV-2 (3.8%) than patients negative for SARS-CoV-2 (8.0%) and those who were not tested for COVID-19 (7.1%). In addition, rates of contamination were higher as coagulase-negative *Staphylococcus* spp were most often seen in inpatients positive for SARS-CoV-2 (59.7%), compared to 32.0% in those negative for SARS-CoV-2 and 29.8% in those who were not tested.^47^ Positive blood cultures due to contamination may have increased antibiotic use and strained hospital resources.

### Radiographs

Chest radiographs and computed tomography (CT) scans play an important role in the diagnosis of COVID-19 pneumonia in hospitalized patients.^48^ A chest radiograph can be negative early in disease and does not rule out the diagnosis.^49^ Chest radiographs may show bilateral ground glass opacities, peripheral linear opacities, or septal thickening.^48–50^ In severe disease, radiographs may demonstrate dense consolidation, particularly in the lower lung lobes. Conversely, radiographs of patients with bacterial pneumonia typically manifest with lobar consolidation, in addition to focal and multifocal consolidation.^50^ Less common findings include ground glass opacities, peribronchial thickening, centrilobular nodular opacities, and pleural effusion. Antibiotics were frequently prescribed for CAP despite lack of characteristic findings for bacterial pneumonia, perhaps because of the myriad presentations of COVID-19.

### Behavioral considerations in prescribing of antibiotics

The COVID-19 pandemic highlights the human desire of HCP to intervene, particularly when a patient is severely ill, which can lead to a suspension of evidence-based medicine (EBM) at the bedside. During a new public health emergency there is significant uncertainty, with an inherent lack of high-quality data, constantly emerging new data, and rapidly changing guidelines. For example, anecdotal data from poorly designed studies resulted in the rapid adoption of hydroxychloroquine for COVID-19 therapies.^51^ Ultimately, higher-quality evidence from multiple randomized trials established hydroxychloroquine was not effective for COVID-19 and in fact was associated with arrhythmia and other adverse effects,^52^ and this practice was abandoned.

As discussed, practitioners frequently have prescribed antibiotics for hospitalized patients with COVID-19, despite increasing evidence that bacterial coinfections are uncommon.^53^ Studies have found that physicians perceive that an antibiotic can prevent clinical deterioration and that they do not identify the potential for adverse events from antibiotic use as influential,^54^ even though antibiotics in this context do not benefit the patient and do increase the risk of adverse drug effects, *Clostridioides difficile* infection, and the emergence of MDROs.^2^ The consequence of increased antibiotic resistance is somewhat abstract and does not counteract the desire to actively intervene for a patient.^55^

To implement strategies to promote appropriate EBM in future public health emergencies, it is important to understand how medical teams make therapeutic decisions and what influences antibiotic prescribing,^56^ especially because it is difficult for clinicians to use clinical knowledge in a setting they perceive to be of extreme risk for their patient.^57^ Diagnostic uncertainty drives antibiotic prescribing, and it is not clear that clinical testing or other data offset the strong societal norm that action (eg, prescribing an antibiotic) is valuable and the action of omission is worse than that of commission.^58^ Physicians tend to follow their colleagues and mimic their practices, for better or worse, and they also frequently defer to the medical hierarchy.^59^ All of these factors have been at play during COVID-19.

## Recommendations

(See Table 2).

## How can facilities and HCP improve antibiotic prescribing during a pandemic or epidemic?

### Recommendation

- HCP should limit initiation of antibiotics – particularly broad-spectrum agents – for patients in acute care settings with high pre-test probability for a viral infection such as SARS-CoV-2 or influenza, even if there is lack of readily available and accurate diagnostics.

### Discussion

There is no evidence that routine antibiotics are needed for respiratory viral pandemics in patients who do not exhibit clear signs of bacterial coinfection. Previous literature, mainly encompassing influenza, has described bacteria in respiratory cultures and autopsy samples and has reported that coinfections are an important cause of morbidity and mortality, necessitating timely diagnosis and antibacterial therapy.^30,53^ Methodologic issues have been noted with the data upon which these conclusions are based, such as inadequate identification of colonization versus infection, and alternate conclusions have emerged that actual bacterial coinfection during influenza outbreaks and SARS-1 was a rare occurrence.^60,61^

During the 2009 H1N1 influenza pandemic, bacterial coinfection was reported in as many as 20% of critically ill patients and in 12% of non-ICU hospitalized patients.^30,53^ However, bacterial coinfection rates are low in patients with COVID-19^31,32^ or other coronaviruses and are not a major factor at the time of admission to the hospital.

A review article of 18 studies of patients with SARS-1, MERS-CoV, SARS-CoV-2, and other coronaviruses found that 42 (31%) of 135 SARS-1 patients had documented bacterial or fungal coinfection, a potentially inflated rate due to a concurrent healthcare-associated outbreak of MRSA.^62^ In a review of 349 critically ill patients with MERS-CoV, only 5 (1%) of 349 patients were documented to have bacterial coinfection on admission.^63^ For other coronavirus infections, bacterial coinfection was observed in 43 (13%) of 331 patients, with no specific bacteria predominating.^62^

With SARS-CoV-2, a meta-analysis of 24 studies with 3,338 patients identified 3.5% of COVID-19 cases had bacterial coinfection on presentation.^53^ Bacterial coinfection was more common in critically ill patients (8.1%). Other studies confirm low rates of community-acquired bacterial coinfection (3.1%–5.5%).^31,32,64^ Another article reviewed 30 studies of 3,834 patients.^30^ Seven percent of patients with COVID-19 were diagnosed with bacterial coinfection, with a higher proportion in the ICU setting (14% vs 4%),^30^ although the study does not provide data on when the infection occurred during the course of the hospitalization.

## What diagnostic testing is appropriate for patients admitted to the hospital?

### Recommendations

- HCP may perform inflammatory marker tests at baseline, particularly in critically ill patients, including C-reactive protein, lactate dehydrogenase, D-dimer, serum ferritin, and high-sensitivity troponin.
- HCP should repeat laboratory testing only to the extent that it provides actionable clinical data.
- HCP can monitor CRP if clinically indicated to inform respiratory viral treatments.
  - For COVID-19, CRP monitoring informed use of biologic agents such as tocilizumab or baricitinib.
- HCP should not use inflammatory markers as the basis for initiation of antibiotics or antifungal agents because they may not be indicative of bacterial or fungal coinfection.
  - HCP should not use procalcitonin routinely to aid in the decision to initiate antibiotics.
- HCP will need to monitor the relationship of inflammatory markers to infection in future infectious epidemics and pandemics.
- HCP should not obtain bacterial cultures or respiratory multiplex PCR tests for patients who do not have indicators consistent with bacterial infection, particularly those with stable clinical status in a non-ICU setting.

### Discussion

Complete blood counts, chemistry, inflammatory markers including CRP, D-Dimer, serum ferritin, lactate dehydrogenase, and high-sensitivity troponin are all markers of a proinflammatory state and are associated with increased mortality.^65^ Elevated inflammatory markers may not be indicative of bacterial or fungal coinfection, and they must be interpreted carefully in the setting of severe viral infection. In COVID-19, these markers can help healthcare providers identify transitions from mild to critical infection and start effective COVID-19 therapy.^66,67^ They should not prompt initiation of antibiotics without other clinical signs and symptoms, radiographic, or microbiologic diagnostic testing. For example, procalcitonin can be abnormal in the absence of bacterial infection.^68^ One study in 2,443 patients with COVID-19 showed that procalcitonin was not able to identify bacterial infection (positive predictive value 17%); however, it had a negative predictive value of 99.3% for bacterial infection, although this may be due to the low number of community-associated infections in the study.^69^ It will be necessary to monitor the relationship of inflammatory markers to infection in future infectious epidemics and pandemics to determine whether patterns similar to that observed during the COVID-19 pandemic are seen.

Bacterial cultures were frequently obtained for patients with COVID-19, even though studies have shown that they have a limited role in the management of noncritical, hospitalized patients.^47,53,62,70^ Sputum colonization or contaminants on blood culture were overinterpreted and inappropriately treated. In the ICU, patients with COVID-19 have a higher rate of bacterial coinfection and those microbiologic studies might have an impact on care.^71–73^ Bacterial multiplex PCR can provide information for de-escalation of antibiotics once initiated but has not been shown to be associated with a change in management in most cases of severe COVID-19.^58,62,74^

## When should antibiotics be considered for patients during respiratory viral pandemics and epidemics?

### Recommendations

- It is important for HCP to identify patients who require empiric antibiotic treatment, despite low rates of coinfection on admission for the COVID-19 and prior respiratory viral pandemics.
- If treated at the time of hospital admission, HCP should prescribe antibiotics recommended in guidelines for CAP for patients presenting from the community with coinfection. Antibiotics for pathogens, such as *Pseudomonas aeruginosa*, seen more typically in healthcare-associated infection, are not recommended.
- HCP may consider coinfection with other viruses, such as influenza. If influenza is diagnosed, addition of therapy, such oseltamivir, may be indicated.
- HCP may consider antibiotics, including broad-spectrum coverage, for patients hospitalized for >48 hours who are at greater risk for HAIs and MDROs, when there are new clinical signs that are consistent with healthcare-associated bacterial or fungal infection.

### Discussion

HCP reasonably may consider empiric antibiotic treatment in instances when a patient has new symptoms (eg, new fever after initial defervescence) and/or changes in lung imaging, signs of production of purulent sputum, dense lobar consolidation on radiograph, or rapid progression to severe COVID-19 requiring intubation and pressor support.^31,32^ Because the bacterial pathogens responsible for CAP often colonize the upper airway and opportunistically infect the lung, it is speculated that these same bacteria should be considered in patients with respiratory virus–related pneumonia including COVID-19 within the first days of hospitalization and guidelines for CAP treatment should be followed.^75^ *Streptococcus pneumoniae* is the pathogen most frequently associated with influenza, followed mainly by *S. aureus,* and *Hemophilus influenzae*.^76^ Recent studies have identified *S. pneumoniae* and *S. aureus* as the main causative agents of community-acquired coinfection at the time of COVID-19 diagnosis.^77^ Data on bacterial coinfections in patients who are immunosuppressed are sparse, but there may be scenarios, such as patients with febrile neutropenia, in which empiric antibiotic therapy at the time of COVID-19 or other respiratory viral diagnosis is justified.^78^

It is important that HCP also consider the possibility of coinfection with viruses other than the pandemic strain. The use of nonpharmacologic interventions (eg, mask use, social distancing) significantly disrupted the transmission of non–SARS-CoV-2 respiratory viruses during the first surge of the COVID-19 pandemic such that influenza activity decreased in March 2020 and remained at historic lows. As these nonpharmacologic interventions are relaxed, HCP should anticipate rising circulation of influenza and other respiratory viruses to include the possibility of coinfection with SARS-CoV-2. In the event of cocirculating influenza and SARS-CoV-2, NIH guidelines suggest HCP initiate empiric oseltamivir pending the exclusion of influenza by nucleic acid detection assay in upper-respiratory-tract specimens for non-intubated patients and in both upper- and lower-respiratory-tract specimens for intubated patients.^79^

Patients who are admitted for >48 hours are at increased risk for HAI, including those that are multidrug resistant. It is reasonable to consider antibiotic choices that include activity against healthcare-associated pathogens such as *P. aeruginosa.* Fungal infection, such as bloodstream infection in patients with prolonged central venous catheters, should also be considered if appropriate.

## What microbiologic and radiographic diagnostic tests are appropriate if antibiotics are started for patients with possible bacterial coinfection?

### Recommendations

- Before initiating antibiotics, HCP should attempt to establish a microbiologic diagnosis.
- HCP should limit respiratory multiplex PCR tests to ICU patients and patients who require broad-spectrum antimicrobial therapy.
- HCP should restrict repeat microbiologic testing to changes in a patient’s clinical status.
- HCP should perform a nasal MRSA swab for patients started on anti-MRSA treatment.
- HCP should not perform routine testing for fungal infection in the absence of a clinical presentation that raises that concern.
- HCP should review the necessity of antibiotics within 48–72 hours, as results from tests become available, and should de-escalate or discontinue antibiotic therapy based on those results and clinical response.
  - Procalcitonin results may aid the de-escalation or discontinuation of antibiotic treatments.
- HCP should obtain chest radiographs to assess the extent of lung involvement, but daily repeat studies are not indicated. Use of CT of the chest should be reserved for circumstances in which results of the CT may result in a change in clinical management (eg, pulmonary embolus).

### Discussion

As noted, HCP should not obtain microbiologic studies for patients without clinical signs and symptoms suggesting a bacterial coinfection. However, in all cases in which antibiotics are started in patients with severe disease (eg, in the ICU) and those requiring broad-spectrum antimicrobial therapy, HCP should obtain relevant samples, for example, sputum cultures and, as appropriate, lower respiratory PCR for a microbiologic diagnosis. Those tests can help HCP appropriately choose definitive therapy or identify opportunities to de-escalate or stop antibiotic treatments. Rapid multiplex PCR can have a role in antibiotic stewardship for critically ill patients^80^ if clinicians are supported by ASPs to interpret and trust those results. Nasal PCR screening for MRSA allows de-escalation for anti-MRSA therapy (eg, vancomycin) in patients with severe CAP and those with risk factors (eg, recent hospitalization, recent receipt of antimicrobial therapy, or prior isolation of MRSA),^43^ and has been shown to have up to 100% negative predictive value, including for hospitalized patients with COVID-19.^81^

Limited data exist on the role of viral and fungal testing to evaluate for coinfection in patients with COVID-19. Routine fungal testing does not lead to a change in medical management^62^ and is not recommended in the absence of a clinical presentation that raises that concern.

HCP should perform ongoing review of the necessity for antibiotics. As test results become available, they should de-escalate or discontinue antibiotics. For HCP planning to treat a patient beyond 5–7 days, studies have suggested PCT as a tool to aid in the discontinuation of antibiotics for community-acquired pneumonia (CAP).^43^ Several retrospective studies assessed the use of PCT alone or in combination with CRP in patients with COVID-19^82–84^ and showed a potential impact on de-escalating or discontinuing antimicrobials.

If healthcare-associated pathogens or MDROs are not identified, HCP should adjust the patient’s antibiotics. Continued signs of inflammation and clinical instability, in the absence of a patient’s response to antibiotic treatment, suggests that COVID-19 is the driver of the clinical picture and antibiotics should be discontinued. De-escalation of antibiotics at 72 hours in the absence of evidence of coinfection, even in the setting of severe sepsis and septic shock, has been well studied and is a safe strategy for the care of patients with viral infections.^85,86^ ASPs can support frontline HCP to stop antibiotics, even in the face of continuing signs of severe illness, with clear guidelines for regular review and cessation of antimicrobial therapy, particularly in the absence of positive bacterial cultures.

HCP should obtain chest radiographs to assess for the extent of lung involvement. Serial chest radiographs are helpful during disease progression but should not be repeated daily.^87^ Chest CT imaging can aid in identification of severe COVID-19 pulmonary complications, ventilation management strategies, and prognosis^88^ and should be reserved for circumstances in which there may be a change in clinical management (eg, pulmonary embolus) because chest CT findings are not able to reliably distinguish COVID-19 from other causes of pneumonia.^89^

## What is the role of the ASP in outbreaks, epidemics, or pandemics?

### Recommendation

- ASPs should monitor emerging information and updates to national and international guidelines that are relevant to antibiotic prescribing, revise clinical recommendations relevant to antimicrobial use, and educate frontline HCP to support appropriate antibiotic stewardship.

### Discussion

ASPs are crucial to improving antibiotic use during a pandemic or epidemic^90,91^ by developing evidence-based treatment guidelines and institutional standards of care. Many of the activities of the ASP during a pandemic or epidemic are extensions of its core functions. ASPs actively monitor patients, identify emerging outbreaks with antibiotic-resistant pathogens, and tailor appropriate antibiotic therapy based on data.^92^ They work with clinicians to provide ongoing review of the appropriateness of antibiotics and can weigh the potential harms such as *C. difficile* infection in the patient and promotion of antibiotic resistance. ASPs provide education and training throughout the institution. During a pandemic, the institution should allocate appropriate resources to make sure that these routine activities can be ramped up to meet the needs of the hospital, provide support for frontline HCP, and improve the care of patients. By supporting clinicians, ASPs can encourage appropriate practices to offset the tendency to default to antibiotic prescribing for critically ill patients.

During a pandemic or epidemic, the ASP can track emerging information relevant to antimicrobial prescribing, including the risk of bacterial or fungal coinfection as a result of a respiratory viral infection or its treatments (eg, dexamethasone, IL-6 inhibitors) and revise clinical recommendations. The ASP can disseminate and adapt new information as more evidence becomes available. The ASP also can educate clinicians to limit initiation of antibiotics in the setting of high pre-test probability for a viral infection such as SARS-CoV-2 or influenza, even if there is lack of readily available and accurate diagnostics. ASPs can provide support for frontline HCP to stop antibiotics, even in the face of continuing signs of severe illness.

ASPs can lead multidisciplinary treatment groups and implement appropriate stewardship interventions. One success to this approach during the pandemic is illustrated by Pettit et al.^93^ At their institution, a COVID-19 management workgroup that included attending physicians from the ICU, internal medicine, immunology, and ID, as well as ID pharmacists evaluated available evidence regarding the use of antibiotics in patients with COVID-19. They developed a diagnostic and treatment guideline and disseminated it throughout the institution. The workgroup facilitated adherence to the guideline with daily virtual rounds with a dedicated COVID-19 ID service and the primary team for all patients admitted with COVID-19. Importantly, the guideline incorporated diagnostic considerations to delineate the syndrome, a typical first step in the antimicrobial reasoning process.^94^ Through this evidence-based intervention, the facility experienced a median decrease of 1.3 days of antibiotic therapy after implementation.

Other examples of successful ASP interventions during COVID-19 included optimization of the electronic record stewardship module to facilitate daily review of COVID-19 patients^95^ and the creation of a clinical guidance team to provide daily, patient-specific antibiotic stewardship recommendations.^96^

Kubin et al^25^ described the multifaceted ways ASPs can improve care through communication, education, inventory control and review of evolving recommendations and new agents. ASPs can adapt their communication strategies during a pandemic (eg, virtual rounds) and work with hospital leadership on optimal strategies for dissemination of new information. Development of clinical guidelines can be shared onsite, and through health system networks and telemedicine,^97^ providing similar benefits to smaller hospitals with less on-site expertise. Importantly, ASPs can frame inappropriate antibiotic prescribing as a low-value intervention and engage HCP in shared decision making.^58^ Many reports have highlighted that targeted stewardship interventions were effective in reducing antibiotic use among patients infected with SARS-CoV-2.^93,98^

ASP efforts during the COVID-19 pandemic have gone far beyond antibiotic management. They have been crucial to assess to emerging treatment regimens for COVID-19, implementing use of remdesivir, dexamethasone, or interleukin-6 inhibitors, and providing expertise and resources to help implement monoclonal antibody treatment and COVID-19 vaccinations.^99^ ASPs have addressed drug shortages and developed strategies to maintain appropriate care.^100,101^ ASPs can be essential in discussions of allocation of scarce resources and the ethical distribution of treatments with limited supply. ASPs can empower hospital leadership and frontline clinicians to address misinformation about emerging therapeutics. It is anticipated ASPs can be as essential in any future infectious disease epidemic or pandemic. Furthermore, ASPs that work closely with infection prevention programs can leverage their skills and experience early in a pandemic.

## Involving ASPs in facility incident management protocols

The process of incident management describes activities done to prepare for, respond to, and learn from crises and emergency events. In the United States and elsewhere, these activities broadly are grouped into the categories of preparedness, mitigation, response, and recovery, and are designed to establish a common operating procedure or picture by which federal, state and territorial, local, municipal, and organizational levels can align efforts.^102,103^ Healthcare organizations adhere to these phases, and these stages are embedded in healthcare protocols. For this reason, the incident management framework can be used to integrate core organizational functions, such as those handled by ASPs, into the emergency preparedness and response activities of their organizations (Table 3).

**Table 3.** The Role of the ASP in Incident Management

Incident Management Phase	Activities of the ASP
**Preparedness** A healthcare organization’s Emergency Management Program (EMP) analyzes how the organization will maintain its core mission when faced with a range of emergencies or threats EMP develops the emergency operations plan (EOP)^105^ EOP details response and recovery plans, the structure of the hospital response, and the execution of the hospital incident command structure (HICS)^106^	EMP engages ASP leadership to obtain input into policies, guidance, and resources for functions of the ASP during crises are contained in the organization’s emergency operations plan (EOP)
**Mitigation** Phase is characterized by efforts aimed to prevent escalation of a crisis and reduce damage by evaluating recommended strategies and seeking consultation with healthcare experts and those involved in internal risk management, eg, ethics specialists, and local and national public health authorities Healthcare organization may activate HICS	Healthcare organization involves the ASP Director, or similar role, in the HICS Healthcare Incident Management Team (HIMT) as a medical-technical specialist to provide input into considerations including Emerging information about nature of the infectious threat and mode of transmission Assessment of supplies of potential therapies and equipment Access to data Need for education for HCP
**Response** Phase encompasses activities that an organization undertakes to manage an emergency or crisis	Healthcare organization continues to engage the ASP Director in HICS in identifying and drafting guidance and care protocols related to antimicrobial use during the emergency
**Recovery** Phase when the organization assesses how to “return to normal” Coordination among the EMP and organizational leaders to: Conduct incident debriefings and after-action reports Identify risks and vulnerabilities that may impede recovery Assess how to improve protocols for future emergencies (106) Evaluate responsibilities absorbed during a crisis Consider revisions to the EOP to address gaps and failures	EMP seeks input from ASP leadership on immediate needs, when and how to scale back activities, and how procedures and policies may be revised In the context of COVID-19, an ASP may have absorbed activities beyond its core functions. Recovery may involve considering how these additional activities should be Incorporated into the ASP’s scope of work, stepped down, and/or included in the EOP Resourced to adequately support them

The incident management framework operates as a cycle^104^; thus, lessons learned from a crisis feed into improving preparedness efforts to address future situations, such as the over-use of antimicrobials as described in this paper and the role of ASPs in managing them.

## Statement limitations and areas for future research

This statement’s recommendations represent a synthesis by the authors of their analysis of the published literature, expert review and consensus, and the authors’ clinical and professional experiences. As stated earlier, this statement is not a substitute for individual judgement of qualified professionals or oversight panels. Users of this document and its recommendations should be aware that it is based on a librarian-conducted search of the published literature and author screening of abstracts and full text; it is not based on a systematic literature review or grading of evidence (see Methods).

There are numerous limitations in the body of evidence that informs this statement, as described throughout, including the role of viral and fungal testing to evaluate for coinfection in patients with COVID-19 and the role inflammatory markers may have in diagnoses of patients infected with emerging pathogens that may contribute to outbreaks, epidemics, and pandemics in the future. This statement advocates that medical teams prepare for future infectious diseases emergencies by improving understanding of how they make therapeutic decisions in the face of evolving evidence and uncertainty, how they involve ASPs in the full scope of incident management efforts (Table 3) in therapeutic decisions during a crisis, and in the context of lessons learned during COVID-19, what changes should occur in policies and practices to prevent inappropriate antibiotic prescribing and further contributions to antibiotic resistance in bacterial and fungal pathogens.

In conclusion, the COVID-19 pandemic has exacerbated the global public health crisis of antibiotic resistance by increasing inappropriate antibiotic prescribing, which was especially common early in the COVID-19 pandemic when HCP were uncertain of the benefit of antibiotics and were concerned with patient deterioration. The practice continued even after it became clear that coinfection with bacteria is uncommon with COVID-19, clinical indicators used to differentiate viral from bacterial infection were no longer valid, and the harms of overuse became evident.

This statement urges facilities and HCP to prevent harm and facilitate evidence-based care by applying lessons learned during COVID-19 to future viral pandemics. This should be done by leveraging the expertise in ASPs to counter inappropriate prescribing practices. During a pandemic, ASPs can lead multidisciplinary teams to compile up-to-date evidence and disseminate care protocols; monitor prescribing and lab assay ordering to detect and mitigate overuse or improper use; and partner with infection prevention and control and incident command partners to integrate and support response efforts. As we rebuild from the COVID-19 experience, ASPs should be supported as a cross-cutting foundation for our public health and healthcare system infrastructures.
